# An *in vitro* potency assay using nicotinic acetylcholine receptor binding works well with antivenoms against *Bungarus candidus* and *Naja naja*

**DOI:** 10.1038/s41598-018-27794-3

**Published:** 2018-06-26

**Authors:** Kavi Ratanabanangkoon, Pavinee Simsiriwong, Kritsada Pruksaphon, Kae Yi Tan, Bunkuea Chantrathonkul, Sukanya Eursakun, Choo Hock Tan

**Affiliations:** 1 Laboratory of Immunology, Chulabhorn Research Institute, Bangkok, 10210 Thailand; 2 Chulabhorn Graduate Institute, Bangkok, 10210 Thailand; 3 Department of Microbiology, Faculty of Science, Mahidol University, Rama 6 Road, Bangkok, 10400 Thailand; 4 Department of Microbiology, Faculty of Medicine, Chiang Mai University, Chiang Mai, 50200 Thailand; 5 Department of Molecular Medicine, Faculty of Medicine, University of Malaya, 50603 Kuala Lumpur, Malaysia; 6 Department of Pharmacology, Faculty of Medicine, University of Malaya, 50603 Kuala Lumpur, Malaysia

## Abstract

In order to facilitate/expedite the production of effective and affordable snake antivenoms, a novel *in vitro* potency assay was previously developed. The assay is based on an antiserum’s ability to bind to postsynaptic neurotoxin (PSNT) and thereby inhibit the PSNT binding to the nicotinic acetylcholine receptor (nAChR). The assay was shown to work well with antiserum against Thai *Naja kaouthia* which produces predominantly the lethal PSNTs. In this work, the assay is demonstrated to work well with antiserum/antivenom against *Bungarus candidus* (BC), which also produces lethal presynaptic neurotoxins, as well as antivenom against Sri Lankan *Naja naja* (NN), which produces an abundance of cytotoxins. The *in vitro* and *in vivo* median effective ratios (ER_50_s) for various batches of antisera against BC showed a correlation (*R*^2^) of 0.8922 (*p* < 0.001) while the corresponding value for the anti-NN antivenom was *R*^2^ = 0.7898 (*p* < 0.01). These results, together with the known toxin profiles of various genera of elapids, suggest that this *in vitro* assay could be used with antisera against other species of *Bungarus* and *Naja* and possibly other neurotoxic snake venoms worldwide. The assay should significantly save numerous lives of mice and accelerate production of life-saving antivenoms.

## Introduction

Snake envenomation is an important yet neglected medical problem in various developing countries with an estimated annual envenomation world wide of about 5.5 million cases^1,2^. Effective and affordable antivenoms (AV) which are the mainstay for treatment remain unavailable in several parts of the world while research efforts are undertaken to solve this problem^3^. In the development and production of an AV, an important step involves the *in vivo* assay to evaluate the potency of the produced antiserum/antivenom. The standard murine lethality neutralizing assay is considered by WHO as an essential AV potency assay. This assay is used to find first, the median lethal dose (LD_50_) that determines the lethality of the venom, and the median effective dose (ED_50_) of the AV^4,5^. The assay is expensive, laborious and, due to biological variation, often give highly variable results. In addition, some murine lethality results might not be consistent with the relevant efficacy outcomes in humans^6,7^. In Thailand, as well as in many Buddhist countries, it is very difficult to find researchers or students who would agree to do these *in vivo* experiments.

Because of these reasons, various *in vitro* assays have been developed to reduce or replace the murine lethality assay. The most widely used assay is based mainly on ELISA^8–10^ but some of these assays have often been shown to give poor correlation with the *in vivo* assay^11,12^. Moreover, the antigen-antibody ‘binding’ reaction of ELISA may not result in the ‘neutralization’ of the antigen, and therefore alternative *in vitro* assay should be developed.

Recently, a novel *in vitro* assay using solubilized, purified nicotinic acetylcholine receptor (nAChR) binding has been developed for AV potency assay against the Thai cobra *Naja kaouthia*^13^. This elapid snake produces mainly postsynaptic neurotoxins (PSNT) which binds specifically and with high affinity to the nAChR at the muscle endplate and thereby inhibits the neuro-muscular transmission causing death in animals. The *in vitro* assay therefore closely mimics the toxicological reactions *in vivo*; and the *in vivo* and *in vitro* potency assays showed a correlation *R*^2^ = 0.9807, p < 0.0001. The assay should also work well with AVs against elapids in the genera other than *Naja* which produce mainly or exclusively post-synaptic toxins (PSNTs) e.g., King cobra, *Ophiophagus hannah*^14^.

However, snakes in the genus *Bungarus* (kraits) also produce, in addition to PSNTs, the lethal presynaptic neurotoxins (β-neurotoxins) which in elapids, belong to the group 1 phospholipase A_2_ enzymes. These toxins do not bind to nAChR but react with receptors on the membrane of the motor nerve terminals which contain the acetylcholine vesicles. The toxins hydrolyze the phospholipids of the plasma membrane, resulting in the loss of synaptic vesicles in the nerve terminal. Eventually the nerve terminals degenerate with the failure of the neuromuscular transmission^15^. The LD_50_ of the β-neurotoxins is about 10 ng/g^16^ which is considerable lower than that of the PSNT (0.18 μg/g mouse)^17^. It is therefore conceivable that in the case of some *Bungarus* venoms, death could be caused, at least in part, by the β-neurotoxins; but this effect would not be measured by the nAChR binding of the *in vitro* potency assay. Consequently the developed *in vitro* assay might not be useful for assay of AV against *Bungarus* spp.

Another interesting case is the *Naja naja* (Indian cobra) which is a WHO category 1 medically most important elapid in India, Pakistan and Sri Lanka. Envenomation by this snake resulted in muscle weakness and death by respiratory failure which is likely the effect of the venom PSNTs. Interestingly, the venom of the Sri Lankan snake was shown by proteomics study to contain 71.55% of cytotoxins (cardiotoxins)^18^. These three finger toxins (3FTs) cause severe local tissue necrosis in most (88%) of the victims^18^ and could possibly contribute to the venom lethality. It is therefore interesting to investigate whether the developed *in vitro* nAChR binding assay could be used for potency assay of AV against this cobra.

We report here that the *in vitro* nAChR binding assay, when used in the potency determinations of AVs against *Bungarus candidus* (Thailand) and *Naja naja* (Sri Lanka), gave high correlation with the corresponding *in vivo* murine lethality neutralization assays.

## Results

### Studies on the optimal conditions of the *in vitro* AV potency assay

#### The optimal concentrations of *NK3*, nAChR, rat anti-nAChR antibody and goat-anti-rat HRP conjugate used in the assay

The optimal concentrations of these four reagents used in the *in vitro* potency assay were described in a previous study^13^. The optimal concentration of NK3 for coating the plates was 15 µg/ml, and 0.707 µg/ml of nAChR for binding to the NK3 coated plate. Rat anti-nAChR serum and goat anti-rat-IgG conjugated HRP were used at 1:1600 dilution and 1:4500 dilution, respectively.

#### Inhibition of nAChR binding to *NK3*-coated plate by *B. candidus* or *N. naja* venom

**Figure 1:**
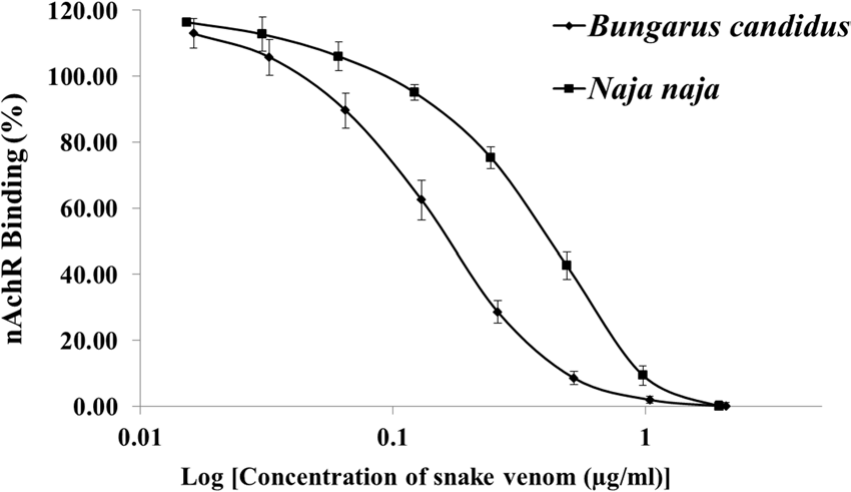
Inhibition of nAChR binding by *B*. *candidus* and *N*. *naja* venoms to NK3-coated plate. Data were means ± SD of 3 determinations.

Crude *B*. *candidus* and *N*. *naja* venoms were separately used to determine the 50% inhibition of the nAChR binding (*in vitro* IC_50_). In the first step, various concentrations of crude *B*. *candidus* (or *N*. *naja*) venom and the purified nAChR (0.707 µg/ml) were incubated for 1 h at 25 °C. After the incubation, the solution was added to the NK3 coated plates. The concentration of the venom that blocked the binding of nAChR by 50% was the IC_50_. The results (Fig. 1) showed that the IC_50s_ of *B*. *candidus* and *N*. *naja* venoms were 0.1625 ± 0.0172 µg/ml and 0.4067 ± 0.0292 µg/ml, respectively.

#### Neutralization of *B. candidus* or *N. naja* venom by horse monospecific antisera against *B. candidus* or by Vins antivenom against *N.naja* as determined by nAChR binding to the *NK3*-coated plate

**Figure 2:**
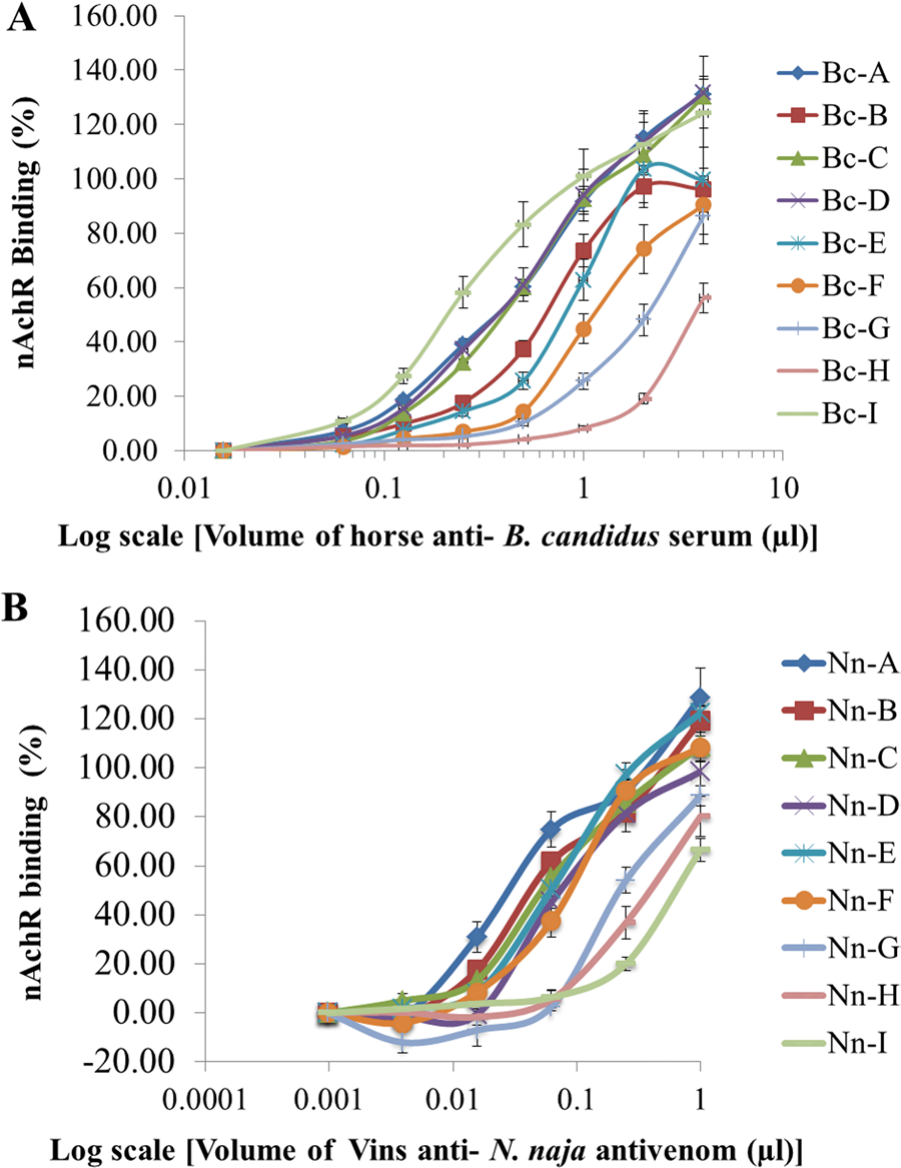
Neutralization of *B*. *candidus* venom by horse anti-*B*. *candidus* sera (**A**) and neutralization of *N*. *naja* venom by Vins anti-*N*. *naja* antivenom (**B**) as determined by nAChR binding to NK3-coated plate. The results were means ± SD of 3 determinations.

Nine horse monovalent anti-*B*. *candidus* sera were 2-fold diluted from 1:500 to 1:512,000. These diluted sera of different horses were separately incubated with 5xIC_50_ of *B*. *candidus* venom (1.4029 µg/ml) in the ‘Pre-incubation 1′ experiment. The reaction mixtures were ultrafiltered and subjected to ‘Pre-incubation 2′. The resulting reaction mixtures were then added to the NK3-coated plates. The nAChR binding to the plate was read at OD_450nm_ and the results which were expressed as % nAChR binding are shown in Fig. 2A. The corresponding results for the *N*. *naja* venom and the Vins anti-*N*. *naja* antivenom are shown in Fig. 2B.

**Figure 3:**
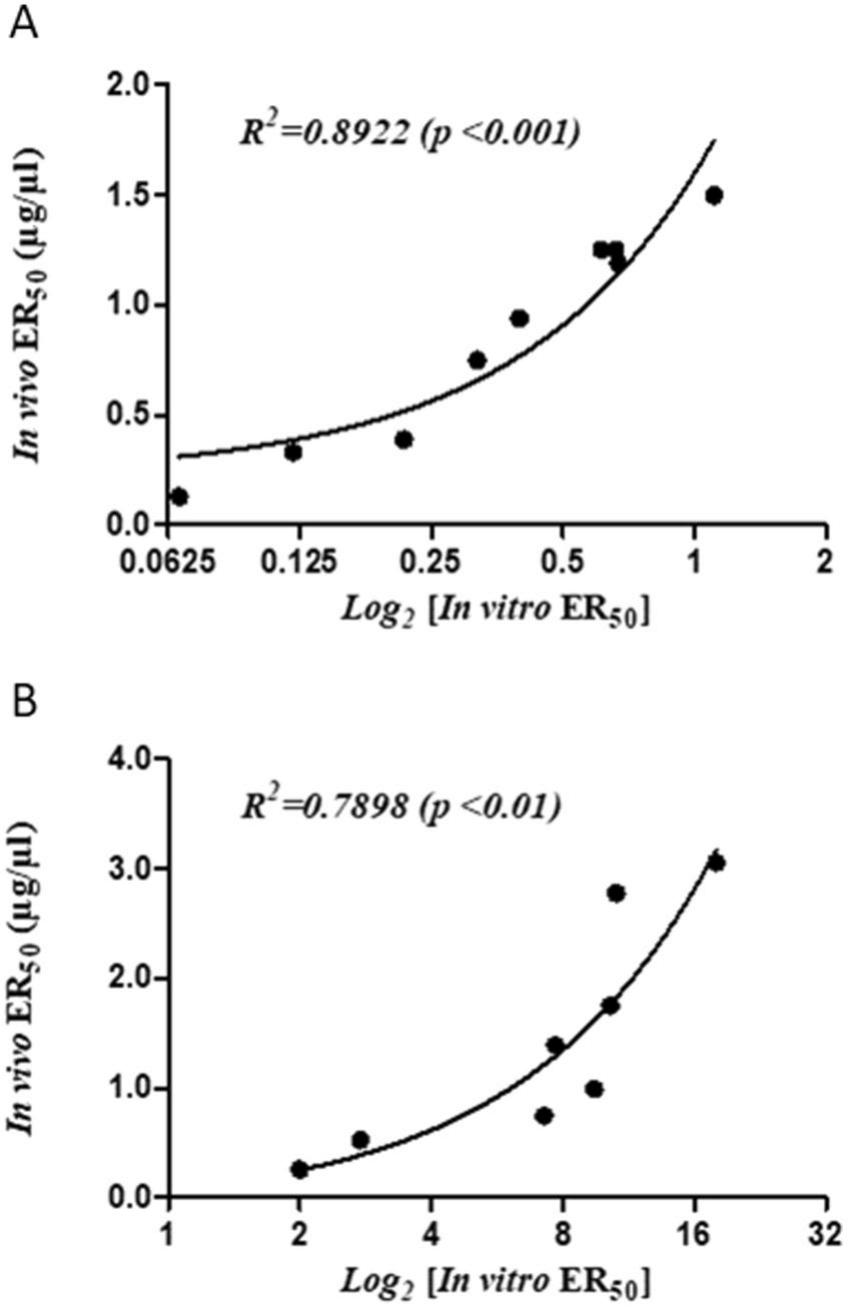
The correlation between the *in vitro* and *in vivo* ER_50_s of the anti-*B*. *candidus* sera (**A**) and of the anti-*N*. *naja* antivenom (**B**).

The *in vitro* ER_50_s, expressed as µg of *B*. *candidus* venom neutralized per µl of antiserum, and the *in vivo* ER_50_s (mg venom/ml antiserum) of the 9 horse anti-*B*. *candidus* sera are shown in Table 1. The correlation between the *in vitro* ER_50_s and the *in vivo* ER_50_s of the anti-*B*. *candidus* sera as shown in Fig. 3A was *R*^2^ = 0.8922 (*p* < 0.001).

**Table 1:** *In vitro* and *in vivo* ER_50_s of horse anti-*B*. *candidus* sera.

Number	Horse sera^#^	*In vitro* ER_50_ ± SD (µg venom/µl antiserum)	*In vivo* ER_50_ (mg venom/ml antiserum)
1	Bc-A	0.6698 ± 0.1091	1.19 (1.59–1.86)
2	Bc-B	0.3982 ± 0.0397	0.94 (0.62–1.46)
3	Bc-C	0.6131 ± 0.0203	1.25 (0.83–1.94)
4	Bc-D	0.6597 ± 0.0639	1.25 (0.83–1.94)
5	Bc-E	0.3184 ± 0.0690	0.75 (0.50–1.17)
6	Bc-F	0.2161 ± 0.0230	0.39 (0.26–0.60)
7	Bc-G	0.1206 ± 0.0170	0.33 (0.22–0.51)
8	Bc-H	0.0662 ± 0.0027	0.13 (0.09–0.20)
9	Bc-I	1.1083 ± 0.2009	1.50 (1.00–2.33)

^#^Bc-A to Bc-I were different horse anti -*B candidus* sera.

The *in vitro* and *in vivo* ER_50_s of the horse anti-*N*. *naja* antivenom are shown in Table 2. The correlation between the *in vitro* ER_50_s and the *in vivo* ER_50_s of the anti-*N*. *naja* antivenom as shown in Fig. 3B was *R*^2^ = 0.7898 (*p* < 0.01).

**Table 2:** *In vitro* and *in vivo* ER_50_s of horse anti-*N*. *naja* antivenom.

Number	AV against *N*. *naja* venom sample^+^	Vins AV concentration factor*	*In vitro* ER_50_ ± SD (µg venom/µl AV)	*In vivo* ER_50_ (µg venom/µl AV)
1	Nn-A	3x	17.9322 ± 4.3182	3.06 (2.01–4.66)
2	Nn-B	2.5x	10.5775 ± 2.5381	2.78 (1.83–4.23)
3	Nn-C	1.75x	10.2668 ± 3.3805	1.75 (1.15–2.66)
4	Nn-D	1.25x	7.6916 ± 1.3811	1.39 (0.91–2.11)
5	Nn-E	1x	9.4299 ± 1.2930	0.99 (0.65–1.51)
6	Nn-F	0.75x	7.2445 ± 1.8840	0.75 (0.49–1.14)
7	Nn-G	0.5x	2.7484 ± 0.5063	0.53 (0.35–0.80)
8	Nn-H	0.25x	1.9976 ± 0.5124	0.26 (0.17–0.40)
9	Nn-I	0.125x	1.2335 ± 0.4311	ND

^**+**^Nn-A to Nn-I were Vins horse antivenom against *N naja* at different dilutions.

*1 × represents the AV concentration when one ampoule of AV was reconstituted in 10 ml of distilled water giving a protein concentration of 69.87 mg/ml.

ND: Not determined (antivenom volume used is higher than the maximum indicated volume for mice injection which is 250 µl).

## Discussion

The *in vivo* potency assay is an important step in antivenom development and production. Because of the complexity of the snake venoms, which contain various diverse pharmacologically active proteins, different pre-clinical assays are needed to test antivenom efficacy against the various components. The gold standard of antivenom efficacy tests is the murine lethality neutralization assay, which is the only ‘Essential test’ included in the *WHO Guidelines on Antivenoms for Production*, *Control and Regulation of Snake Antivenom Immunoglobulins*^4^. Depending on the pharmacological profile of a given venom, several other supplementary ‘Recommended tests’ have been proposed e.g., neutralization assays of hemorrhagic, nephrotoxic, myotoxic, neurotoxic, edema-forming, dermonecrotic and measurement of *in vitro* coagulant and defibrinogenating activities^4^.

However, these assays, especially the ‘Essential’ murine lethality neutralization assay, which involves the killing a large number of animals, are faced with various experimental, ethical, religious and regulatory difficulties. Consequently, many *in vitro* assays have been developed, mostly based on ELISA formats, to be used in place of, or to reduce the use of, *in vivo* murine assays. However, because ELISA involves the binding of antivenom antibodies to venom proteins whose toxicity might not be neutralized, results of these *in vitro* assays usually show poor correlation with results from the murine lethality neutralization assay. We have recently developed a novel *in vitro* assay^13^ based on nAChR binding to determine the potency of antivenom against *Naja kaouthia*, an elapid that produces, almost exclusively, postsynaptic neurotoxins that are responsible for death in victims. This assay estimates the binding of venom postsynaptic neurotoxins to nAChR, which is the toxicological reaction involved in the lethal effects of cobra envenomation. In this study, it is shown that the *in vitro* assay should be suitable for the evaluation of horse antisera against *Bungarus candidus*, which produces clinically relevant lethal presynaptic neurotoxins (β-neurotoxins) in addition to PSNT. Moreover, the assay could also be used to evaluate commercial F(ab’)_2_ antivenoms against *Naja naja* (Sri Lanka), which produces very high levels of cytotoxins in its venom^18^.

*Bungarus candidus*, also known as the Malayan krait, is a WHO Category 1 medically important snake in Southeast Asian countries. The venom of this snake contains β-neurotoxins that are highly lethal, in addition to PSNTs^19,20^. These toxins are also produced by several other krait species. They have molecular weights (MW) ranging from 18–22 kDa and are heterodimers with one subunit exhibiting phospholipase A_2_ activity^21^. They do not bind to nAChR; however, they cause neuromuscular blockade leading to death by respiratory failure^16^. The venom of *B*. *candidus*, (Thailand), contains about 26.9% long and short 3FT of subtype PSNT and about 13.5% of β-neurotoxins (CH Tan, unpublished data).

In the present study, it was shown that the *in vitro* nAChR binding potency activity of antisera against *B. candidus* venom was highly correlated with the murine *in vivo* lethality neutralization assay results (*R*^2^ = 0.8922, *p* < 0.001). Since the *in vitro* assay measures postsynaptic neurotoxin binding, and therefore does not detect the clinically important binding of β-neurotoxin to nAChR, it is of interest to know the reason behind the observed high correlation between the results of this assay and those of the *in vivo* lethality neutralization assay when evaluating *B*. *candidus* antisera. This observation may be due to the experimental conditions used for the *in vivo* lethality neutralization assay described in WHO Guidelines^4^. When the mice were challenged with 5xLD_50_ of the venom, the smaller (7–8 kD) faster acting postsynaptic neurotoxin rapidly inhibits the nAChR at the muscle endplate resulting in neuromuscular blockage and death in mice. The β-neurotoxin, which is larger (18–22 kD) and slower acting, has to undergo a number of steps before finally depleting the acetylcholine store of the synaptic vesicles to completely inhibit the neuromuscular transmission. Thus, in the lethality assay (LD_50_ determination), death was likely due to the action of postsynaptic neurotoxins before the action of β-neurotoxins took effect. In the *in vivo* ED_50_ determination, when the antiserum antibodies were added to react with the venom, the antibodies most likely neutralized both the postsynaptic and β-neurotoxins. This is because the β-neurotoxins are larger and has a more complex chemical structure than the postsynaptic neurotoxins. Consequently, the β-neurotoxin should be more immunogenic and have more antibodies against it in the antisera. Thus, under the *in vivo* lethality neutralization assay, postsynaptic neurotoxin plays the major role in determining the lethality neutralization activity. Therefore, the high correlation between the two assays could be due to the fact that both the *in vivo* and *in vitro* assays primarily measured the activity of the postsynaptic neurotoxins. If this is in fact the case, then the murine lethality neutralization assay, when applied to *B*. *candidus* antivenom, is another example of an antivenom potency test where the clinically relevant toxin in human envenomation is not measured^6,7^.

Thus, in the development and production of antivenom against *B candidus*, it may be necessary to perform an additional ‘neurotoxicity neutralization assay’ to determine the neutralization activity for the β-neurotoxin in the venom. This can be done according to the WHO ‘Recommended antivenom efficacy test’ guidelines, which involve the use of isolated chick biventer cervicis nerve-muscle preparations and/or mouse hemidiaphragm phrenic nerve preparations^4^. This *ex vivo* assay should also shed light on the inhibition of β-neurotoxin in the present *in vivo* assay. However, the tests are difficult to perform, require costly equipment and expert technological help and are unlikely to be practicable for most laboratories. Because of the technical difficulty involved in the neurotoxicity assay, WHO Guidelines^4^ concluded that the murine lethality neutralization assay is a reliable assay in predicting the neutralization of neurotoxicity of venoms.

*Naja naja*, i.e. the Indian cobra, is also a WHO Category 1 medically important snake in South Asia. The percent abundances of α-neurotoxins were only 4.8% and 8.91% in *N*. *naja* (India) and *N*. *naja* (Sri Lanka) venoms, respectively, while the cytotoxins were the most abundant venom 3FT subtype in both *N*. *naja* (India) (69.34%) and *N*. *naja* (Sri Lanka) (71.55%)^18^. The cytotoxins, also known as cardiotoxins, are polypeptides with a MW of about 6–7 KDa, and are found only in cobra venom^22^. At lower concentrations, cardiotoxins increase the heart rate and, at higher concentrations, kill the animal by inducing cardiac arrest^23^. Thus the abundant cytotoxins in *N*. *naja* venom might contribute to lethality in the *in vivo* assay. Since the *in vitro* nAChR assay estimates only the nAChR binding of the α-neurotoxins, it is possible that this difference could affect the correlation between the *in vivo* and *in vitro* potency assays and limit the usefulness of the assay for *N*. *naja* antivenom. However, it was shown in the present study that for the antivenom against *N*.*naja* (Sri Lanka), a good correlation between the *in vitro* nAChR-PSNT binding activity and the *in vivo* murine lethality neutralization activity was obtained. The reason behind this observation could be that the presence of a large amount of cytotoxins in the venom did not affect the results of the *in vitro* nAChR - PSNT binding assay since the assay has been shown to be specific to PSNT and not to cytotoxins^13^. Furthermore, the results suggested that death is mainly the result of the α-neurotoxins since α-neurotoxins are about 10 times more toxic, with an LD_50_ of *ca*. 0.1 μg/g in mice while that of the cytotoxins is about 1.0–1.5 μg/g^24,25^.

The observations that the *in vitro* potency assay based on nAChR-PSNT binding could be used to evaluate antivenoms against *B*. *candidus* and *N*. *naja* may have important implications for the use of this assay to assess antivenoms against other elapids. For example, *Micrurus nigrocinctus*, the Central American coral snake, is a medically important snake which has been shown in proteomics studies to contain a variety of toxins. These included several isoforms of short-chain α-neurotoxins as well as muscarinic-like toxins and proteins with similarity to long-chain κ-2 bungarotoxin while the α-neurotoxins predominated^26,27^. Alape-Giron *et al*.^28^ have shown that various venom proteins of *M*. *nigrocinctus* specifically bind to the *Torpedo* spp. acetylcholine receptor while the majority of PLA_2_ fractions exhibit low lethality. The predominant effects of the *M*. *nigrocinctus* venom are neurotoxicity and myotoxicity with death from the venom to be due to respiratory failure caused by the α-neurotoxins. The α-neurotoxins of this venom play a leading role in the lethality of this venom^27,28^. Thus, it is highly likely that the present *in vitro* assay could give high correlation with the *in vivo* murine lethality neutralization assay, and could be used for antivenom potency assays for this snake.

Another interesting group of elapids are the *Dendroaspis* spp. For example, *Dendroaspis polylepis*, an African elapid known as the black mamma, is an extremely deadly snake. The venom of this snake contains postsynaptic neurotoxins^29^ and also dendrotoxins, which are unique to the genus *Dendroaspis*^30^. Dendrotoxins are Kunitz-type proteinase inhibitors. They block the voltage-dependent potassium channels resulting in the facilitation of the release of neurotransmitters at the presynaptic nerve terminal^31^. The α-neurotoxins in *Dendroaspis polylepis* venom are the most toxic proteins in the venom, followed by dendrotoxins. Death due to black mamba envenomation is caused mainly by the postsynaptic neurotoxins, while the less toxic dendrotoxins probably play a secondary role in the lethal action of the venom^31,32^. Based on this information, it is quite likely that the present *in vitro* potency assay will be applicable to the antisera against *D*. *polylepis* as well. A similar conclusion could probably be made with *Hydrophis schistosus* (sea snake) and *Laticauda colubrina* (sea krait) that produce mainly short-chain and long-chain α-neurotoxins, respectively, which have been shown to cause paralysis and death in animals^33,34^. If these predictions are shown to be the case experimentally, the assay is likely to be useful for potency assays of antivenoms against most elapids of the world.

The Vins antivenom against *N*. *naja* used in this study was a F(ab′)_2_ antivenom. ‘Pre-incubation 1′ of the *N*. *naja* venom with the F(ab′)_2_ antibody was followed by ultrafiltration using a membrane with a MWCO of 50kD, instead of the customary 100 kD membrane^13^. This was done to remove the bound and free F(ab′)_2_ antibody, which has a molecular weight of about 100 kDa.

The ability to use the F(ab′)_2_ as well as IgG antibody in the *in vitro* assay is more advantageous compared to ELISA. In ELISA where a secondary antibody is used, different signal intensity will be obtained when the first antibody is a F(ab′)_2_ or an IgG. Of the two, use of an IgG will result in more secondary antibody binding (to the Fc portion) resulting in the generation of a relatively stronger color signal. In ELISA potency assays, where some samples consisted of IgG and some with F(ab′)_2_ antivenoms, comparison between the results may not be possible due to the above consideration. However, this difference in the antibody chemical structures should not be a problem in the present nAChR-PSNT binding assay.

An interesting aspect of this study was that the Vins antivenom is polyspecific against 2 vipers and 2 elapids. Therefore, the antivenom also contained F(ab′)_2_ antibody against the viper proteins but the ‘heterologous’ antibody did not seem to interfere with the nAChR assay as shown by the good correlation result observed. This *in vitro* assay, unlike most ELISA, which employ crude venoms as the antigens and thus often show cross reactivity of the heterologous antibodies with other venom proteins, gave more precise results as would be expected from the highly specific binding of α-neurotoxin to nAChR.

The present *in vitro* potency assay should be very useful in the development and production of effective antivenoms against elapid snakes which, in various countries, are the most medically important cause of snakebite morbidity and mortality. In the development of new antivenoms, whether traditional serum based or synthetic^35^, potency assays of the products have to be carried out. The lethality neutralization assay is an essential pre-clinical test for an antivenom. The present *in vitro* assay could be used to reduce, or in some cases replace, the *in vivo* assay during AV development. Even with the final antivenom product, which must be assayed using the murine lethality assay^36^, the *in vitro* assay could be employed in dose range determination and consequently could reduce the use of mice in this trial and error process.

The nAChR-PSNT binding assay is simple, less variable, less time-consuming and less expensive and could facilitate the development of effective and wide-paraspecific ‘universal’ antivenoms. This would ultimately reduce the mortality and morbidity of snake envenomation, which occurs mostly in poor countries/regions of the world^37^.

## Materials and Methods

The venom of *Bungarus candidus* which was from a pool of several adult snakes of Thai origin, and the horse monovalent antisera against *B*. *candidus* were purchased from Queen Soavabha Memorial Institute (QSMI). Lyophilized *Naja naja* (Sri Lanka) milked from a large pool of adult snakes was obtained from Dr C. Ariaranee. The polyspecific equine lyophilized F(ab′)_2_ antivenom was from Vins Bioproduct Limited, Hyderabad, India; it was prepared against saw-scaled viper (*Echis carinatus*), Indian cobra (*N*. *naja*), Russell’s viper (*Daboia russelii*) and common krait (*Bungarus caeruleus*), Batch no. 01AS12041, Exp date 03/2018. *Naja kaouthia* postsynaptic toxin 3 (NK3) was purified according to Karlsson *et al*.^38^. All other reagents were from Sigma Chemical, St Louis. Missouri, unless stated otherwise. nAChR from *Torpedo californica* electroplaque was purified and the anti-nAChR antisera was generated in rats as described by Ratanabanangkoon *et al*.^13^.

### *In vivo* neutralizing activities of horse monospecific antisera against *B*. *candidus* venom and F(ab′)_2_ antivenom against *N*. *naja* venom

The median lethal doses (LD_50_s) of *B*. *candidus* and *N*. *naja* venoms, were determined by intravenous route as described previously^17^

Neutralization of lethality in mice was carried out according to Tan *et al*.^39^ with slight modification from Ramos-Cerrillo *et al*.^40^. Briefly, a challenge dose (higher than LD_100_) of the venom constituting 5xLD_50_ for *B*. *candidus* (or 2xLD_50_ for *N*. *naja*) in 50 μl saline was pre-incubated at 37 °C for 30 min with varying dilutions of the horse antisera/antivenom in normal saline, to give a total volume of 250 μl.

The venom-antiserum or venom-antivenom mixture was then injected into the caudal vein of mice (n = 4–5, 20–25 g). Food and water were provided *ad libitum*. The results (dead/alive) after 12, 24, 36 and 48 h were recorded. The median effective dose (ED_50_) was determined as the volume of antiserum (µl) that protected 50% of the challenged mice tested. The *in vivo* median effective ratios (ER_50s_) of the antiserum and antivenom were also calculated as described by Morais *et al*.^41^.

### The *in vitro* neutralization potency assays of antisera against *B*. *candidus* and Vins antivenom against *N*. *naja* using nAChR-PSNT binding assay

#### Optimal concentrations/conditions of nAChR, rat anti-nAChR antibody and goat-anti-rat HRP conjugate used in binding to *NK3* coated plate

The format of the basic assay for binding of purified nAChR to the NK3 immobilized microtiter plate was performed as described previously by Ratanabanangkoon *et al*.^13^. Optimal concentrations of NK3, nAChR, rat anti-nAChR antibody and goat-anti-rat-HRP conjugate were established^13^ and used in the experiments that followed. The IC_50_ values were reported as mean ± standard deviation based on findings from three independent experiments.

#### Inhibition of nAChR binding to the *NK3* coated plate by *B*. *candidus* or *N*. *naja* venom

The ability of *B*. *candidus* and *N*. *naja* venoms to individually inhibit the binding of nAChR to NK3 coated plate were expressed as IC_50_ (venom concentration inhibiting 50% of the nAChR binding or the median inhibitory concentration). In this experiment, *B*. *candidus* or *N*. *naja* venom at various concentrations were pre-incubated (25 °C, 1 hr) with a fixed and optimal concentration of nAChR. The mixture was then added to the NK3-coated plate and incubated at 25 °C for 1 h. This was followed by additions of rat anti-nAChR serum at 1:1,600 dilution and incubated at 25 °C for 1 h. Thereafter, 1:4,500 diluted goat-anti-rat-HRP conjugate (Abcam^®^) was added and the mixture was incubated for 60 min at 25 ^o^C. A parallel experiment, in which purified NK3 was used as the reference standard in place of the venom, was carried out. The concentration of the venom used in the pre-incubation experiment that blocked 50% of the nAChR binding to the immobilized NK3 was the IC_50_ of that venom.

#### Inhibition of the *B*. *candidus* or *N*. *naja* venom binding to nAChR by horse antisera against *B*. *candidus* or Vins F(ab′)_2_ antivenom against *N*. *naja*

The horse antiserum/antivenom potency (*in vitro* ED_50_) was determined using the above described format. Varying amounts of horse sera (0.94 nl – 0.96 µl) were pre-incubated with a fixed amount (5xIC_50_) of *B*. *candidus* venom for 1.5 hr at 37 °C in a total volume of 480 µl. This was called ‘Pre-incubation 1′. The antibody-toxin complexes together with the free antibodies were removed from the mixture by filtration through a 100 kDa MWCO ultrafiltration membrane (Amicon^®^). The free venom PSNTs and β-neurotoxins in the filtrates (126 µl) were then allowed to react with an optimal amount of nAChR (14 µl) at 25^o^C for 1 hr; this was called ‘Pre-incubation 2’. The mixtures were transferred to the NK3 coated microtiter wells, followed by the rat anti-nAChR antibody, goat-anti-rat HRP conjugate, etc. The reaction products were then measured. Background control consisted of wells containing non-immune horse serum instead of antisera.

For the *N*. *naja* venom and Vins F(ab′)_2_ antivenom pair, similar and parallel experiment was carried out. In this case, 3xIC_50_ of crude *N*. *naja* venom was incubated with various dilutions of the Vins antivenom and the reaction mixture from the ‘Pre-incubation 1′ was ultrafiltered using a 50 kDa MWCO membrane (Amicon^®^). The filtrate was then incubated with nAChR in the ‘Pre-incubation 2′ and processed as described above.

The percentage of nAChR binding was calculated from the following equation:

1 $$ \% \,{\rm{nAChR}}\,{\rm{binding}}=\frac{({\rm{OD}}\,{\rm{sample}}-{\rm{OD}}\,{\rm{Ag}}\,{\rm{control}})\times 100}{({\rm{ODmax}}-{\rm{OD}}\,{\rm{Ag}}\,{\rm{control}})}$$

‘OD max’ were the binding of nAChR (optimal amount) without pre-incubation with the venom or antiserum.

‘OD Ag control’ were the binding of nAChR after pre-incubation with 5xIC_50_ of *B*. *candidus* venom (or 3xIC_50_ of *N*. *naja* venom) and without antiserum/antivenom in ‘Pre-incubation 1′.

‘OD sample’ were the binding of nAChR to the NK3 immobilized plate after the nAChR (optimal amount) was pre-incubated with filtrate from ‘Pre-incubation 1′.

The dose–response curves of horse sera volumes versus percent of nAChR binding were then constructed. The *in vitro* neutralizing activities (ED_50_s) were the volumes of horse antiserum at which the nAChR binding was blocked by 50 percent compared to wells incubated with non-immune horse sera in place of antisera. The *in vitro* median effective ratio, ER_50_, represented µg venom/µl antiserum that inhibited 50% of nAChR binding was calculated.

Similar manipulation and calculations were carried out with the results obtained from the Vins F(ab′)_2_ antivenom and *N*. *naja* venom pair.

### Ethics approval

The protocol of animal study on mice followed the Council for International Organizations of Medical Sciences (CIOMS) guidelines and was approved by the Institutional Animal Care and Use Committee (IACUC) of the University of Malaya (Ethical clearance No. 2014–09–11/PHAR/R/TCH).

### Miscellaneous procedures

The concentration of protein was determined as described by Lowry *et al*.^42^ and by BCA Protein assay Kit (PierceTM) using bovine serum albumin as the standard. GraphPad Prism 5.0 program was used to determine the IC_50_ and ED_50_ values. Linear regression with GraphPad Prism 5.0 software was employed in the correlation analysis. Briefly, the correlation coefficient *R* was determined from the linear regression model, and *R*^2^ is the square of the correlation coefficient. An *R*^2^ of 0.8–1.0 indicates that the regression line fits well the data in correlation. The statistical significance of the correlation test was set at *p* < 0.05.
